# Integrated single-cell transcriptomic and machine-learning analyses identify a CD36-associated macrophage state in diabetic nephropathy

**DOI:** 10.3389/fimmu.2026.1868468

**Published:** 2026-08-20

**Authors:** Xin Yang, Jiangying Li, Ying Zeng, Gao Wang, Jilan Zeng, Dehui Liu

**Affiliations:** 1 Department of Nephrology, Ganzhou People’s Hospital, Ganzhou, Jiangxi, China; 2 Department of Pathology, The First Affiliated Hospital, Jiangxi Medical College, Nanchang University, Nanchang, Jiangxi, China; 3 Department of Nephrology, The First Affiliated Hospital, Jiangxi Medical College, Nanchang University, Nanchang, Jiangxi, China; 4 Nanchang University Fuzhou Medical College, Fuzhou, Jiangxi, China

**Keywords:** CD36, diabetic nephropathy, lipid metabolism, lipid-associated inflammation, macrophages

## Abstract

**Background:**

Diabetic nephropathy (DN) is the leading cause of end-stage renal disease worldwide and is characterized by progressive glomerulosclerosis, tubulointerstitial fibrosis, and a chronic inflammatory microenvironment. Although macrophage infiltration and immune dysregulation are recognized as central pathological features, the cell-type-specific transcriptional programs that drive inflammatory amplification in DN remain incompletely understood, particularly at single-cell resolution.

**Objective:**

To identify macrophage-associated transcriptional biomarkers and delineate intercellular communication networks in DN using an integrated workflow combining urine single-cell RNA sequencing, multi-algorithm machine learning, and tissue-level experimental validation.

**Methods:**

Urine scRNA-seq data from 17 healthy-control libraries (GSE157640 and GSE165396) and 8 diabetic nephropathy (DN) libraries (GSE266146) were processed using Seurat and integrated using Harmony. IL6-JAK-STAT3 pathway activity was quantified using AUCell. The GSE30122 cohort was stratified by disease status and randomly divided into training and held-out internal validation sets. Candidate-gene selection using LASSO, random forest, and Boruta was performed exclusively in the training set, and the discriminatory performance of the consensus genes was subsequently evaluated in the held-out internal validation set using receiver-operating-characteristic analysis. Cell-cell communication was computationally inferred using CellChat. Renal CD36 expression and its spatial association with F4/80-positive macrophages were assessed in an STZ-induced mouse model.

**Results:**

Single-cell analysis identified major epithelial and immune cell populations in the urine of patients with DN and controls. IL6-JAK-STAT3 pathway activity was significantly elevated in DN, with macrophages showing the highest enrichment. Machine learning identified CD36 as a consensus biomarker with an AUC of 0.894 in the external validation cohort. CD36 was predominantly expressed in macrophages, and CellChat analysis predicted inflammatory and profibrotic ligand–receptor communication patterns associated with CD36-detected macrophages. In DN mice, CD36 was significantly upregulated at both the mRNA and protein levels and co-localized with F4/80-positive macrophages in renal tissue.

**Conclusions:**

CD36 represents a macrophage-enriched candidate molecular marker associated with an IL6–JAK–STAT3-high transcriptional state and predicted intercellular communication patterns in DN. These findings support further investigation of CD36, although its clinical utility and therapeutic relevance require independent clinical and functional validation.

## 1 Introduction

Diabetes mellitus has reached epidemic proportions globally. According to the Global Burden of Disease 2021 study, an estimated 529 million people were living with diabetes worldwide in 2021, with a global age-standardized prevalence of 6.1%, and this number is projected to rise to 1.31 billion by 2050. As diabetes remains a major cause of death and disability worldwide, these trends underscore the urgent need to better understand the organ-level consequences of chronic metabolic dysregulation (1).

Diabetic nephropathy (DN) is a leading cause of chronic kidney disease and kidney failure worldwide and remains a major microvascular complication of diabetes (2). DN progresses from microalbuminuria through overt proteinuria to declining glomerular filtration rate and, ultimately, kidney failure. While hyperglycemia-driven oxidative stress and renin–angiotensin–aldosterone system activation contribute to its initiation, accumulating evidence over the past two decades has reframed DN not merely as a metabolic or hemodynamic disorder but fundamentally as a chronic inflammatory disease (2–6).

Macrophage infiltration is a cardinal feature of DN, and macrophages play a central role in disease progression by promoting inflammatory signaling, oxidative stress, and fibrotic remodeling, thereby sustaining a self-reinforcing injury microenvironment. Nevertheless, the precise transcriptional states of macrophages in the DN kidney, and the specific signaling hubs through which they communicate with resident renal cell populations, remain insufficiently characterized at single-cell resolution (3–8).

The IL-6/JAK/STAT3 signaling axis occupies a central position in macrophage-mediated renal inflammation (9). IL-6, secreted by activated macrophages and tubular epithelial cells under hyperglycemic conditions, transduces its signal through membrane-bound and soluble IL-6 receptor complexes coupled to gp130, leading to JAK1/JAK2 activation and STAT3 phosphorylation at Tyr705. Nuclear STAT3 then transcriptionally activates a broad repertoire of proinflammatory and anti-apoptotic target genes, creating a positive feedback loop that sustains macrophage activation and perpetuates renal injury (10). Therapeutic blockade of this pathway has shown promise in experimental models of DN, reinforcing its functional relevance.

CD36, a class B scavenger receptor and fatty acid translocase, is constitutively expressed on macrophages and mediates the uptake of oxidized low-density lipoprotein (oxLDL), long-chain fatty acids, and advanced glycation end products, which are enriched in the diabetic renal microenvironment. In macrophages, CD36-mediated oxLDL internalization drives NLRP3 inflammasome activation and NF-κB-dependent inflammatory gene expression, positioning CD36 as an active mediator rather than a passive marker of inflammatory injury (11). However, its cell-type-specific expression landscape in DN and its role in orchestrating intercellular communication within the renal microenvironment have not been systematically characterized (11–13).

Single-cell RNA sequencing of human urine offers a compelling non-invasive window into the renal cellular microenvironment, circumventing the procedural risks inherent to kidney biopsy (14). Urine contains shed renal and immune cells that reflect ongoing parenchymal pathophysiology, and urinary single-cell transcriptomic studies have demonstrated that shed renal and immune cells can reflect intrarenal pathological processes in several kidney diseases, including lupus nephritis, acute kidney injury, and diabetic kidney disease (15–17). However, large-scale urine single-cell studies integrating machine learning-based biomarker selection and experimental validation in DN have not been systematically performed (15).

The present study addresses these gaps through a multimodal analytical framework comprising: (1) urine scRNA-seq analysis to define the cellular landscape and IL6-JAK-STAT3 pathway activity in DN; (2) consensus biomarker identification by intersecting three independent machine learning algorithms in an external validation dataset; (3) CellChat-based inference of CD36-associated intercellular communication; and (4) *in vivo* experimental validation in a mouse model of DN (18). Together, these analyses identify CD36 as a macrophage-associated inflammatory biomarker and provide a mechanistic framework for understanding immune activation in DN.

## 2 Materials and methods

### 2.1 Study overview

This study consisted of three integrated components: (1) analysis of human urine scRNA-seq data from GEO datasets GSE157640, GSE165396, and GSE266146 to delineate the cellular landscape and IL6–JAK–STAT3 pathway activity in DN; (2) consensus biomarker identification in the bulk transcriptomic dataset GSE30122 by intersecting three complementary machine-learning algorithms; and (3) *in vivo* experimental validation of renal CD36 expression and its spatial association with F4/80-positive macrophages in a mouse DN model.

### 2.2 Single-cell dataset and preprocessing

Human urine scRNA-seq data were obtained from three publicly available Gene Expression Omnibus datasets: GSE157640, GSE165396, and GSE266146. GSE157640 and GSE165396 each contributed one pooled healthy-control library, derived from samples pooled from five and 12 healthy donors, respectively. GSE266146 contributed eight individual DN libraries, including four early-DN and four advanced-DN libraries. The two Control libraries were treated as pooled library-level inputs rather than as 17 independent donor-level samples. Raw count matrices were imported into R (version 4.3) and processed using the Seurat package (version 4.4) (19).

Quality control was performed independently for each library-level input before data merging and integration. Seurat objects were initialized with min.cells = 3 and min.features = 200. Cells were retained when nFeature_RNA was greater than 300 and less than 6,000, nCount_RNA was greater than 500, and the mitochondrial transcript percentage was less than 20%. The same filtering criteria were applied to all 10 library-level inputs, and no upper threshold was imposed on nCount_RNA.

Early and advanced DN samples from GSE266146 were combined into a single DN group for the main single-cell analysis. This strategy was adopted because the primary aim of the study was to identify DN-associated cellular and macrophage-related inflammatory features compared with healthy controls, rather than to define stage-specific transcriptional differences. In addition, the number of retained cells from the early-DN samples was relatively limited after quality control, which could reduce the stability of separate stage-specific analyses.

### 2.3 Dimensionality reduction, batch correction, and clustering

After quality control, 918 cells from the GSE157640 pooled Control library, 879 cells from the GSE165396 pooled Control library, and 2,114 cells from eight individual GSE266146 DN libraries were retained, yielding a merged object of 3,911 cells, including 1,797 Control cells and 2,114 DN cells.

Each of the 10 library-level inputs was normalized using the LogNormalize method with a scale factor of 10,000, and 2,000 highly variable genes were identified using the variance-stabilizing transformation method. A total of 3,000 integration features were selected using SelectIntegrationFeatures after excluding mitochondrial and hemoglobin genes. The data were scaled using ScaleData, with mitochondrial transcript percentage regressed out, followed by principal component analysis using 50 components. Based on inspection of the elbow plot, the first 30 principal components were retained for downstream analysis.

Harmony correction was applied to the PCA embeddings using library identity as the batch variable (20), comprising two pooled Control libraries and eight individual DN libraries. Disease group and DN stage were not used as correction variables. The first 30 Harmony-corrected dimensions were used for shared nearest-neighbor graph construction, Louvain clustering at a resolution of 0.6, and UMAP visualization (19, 20). Integration was qualitatively evaluated using Harmony-corrected UMAP plots colored by DN library identity and Control-dataset source, as well as by disease group (**Supplementary Figure 1**) (20).

### 2.4 Cell annotation

Cell types were manually annotated by integrating cluster-specific marker expression with established canonical marker programs for renal epithelial and immune-cell populations. Macrophage and epithelial lineage programs were evaluated in parallel to resolve clusters with potentially overlapping marker expression. The final cluster assignments, canonical marker panels, and cluster-specific markers are provided in **Supplementary Figure 1**, **Supplementary Table 1**.

### 2.5 AUCell pathway activity scoring

IL6–JAK–STAT3 transcriptional enrichment was quantified using AUCell and the human HALLMARK_IL6_JAK_STAT3_SIGNALING gene set from the Hallmark collection of the Molecular Signatures Database. AUCell rankings were generated from the raw RNA count matrix using AUCell_buildRankings, and AUC values were calculated using AUCell_calcAUC, with aucMaxRank set to 10% of the ranked genes. Genes absent from the count matrix did not contribute to the AUC calculation. AUCell scores were analyzed continuously for Control-versus-DN comparisons. For DN-specific downstream analyses, cells with scores above the median DN AUCell score were classified as AUCell-high (score_UP), whereas those with scores at or below the median were classified as AUCell-low (score_DOWN) (21).

### 2.6 Differential expression and functional enrichment

Differential expression between AUCell-high and AUCell-low DN cells was assessed at the cell level using the Seurat FindMarkers function with a Wilcoxon rank-sum test, min.pct = 0.1, and logfc.threshold = 0.1. Genes with an absolute average log2 fold change greater than 0.25 and a Bonferroni-adjusted P value below 0.05 were considered significant. Spearman correlations were calculated between detected HALLMARK_IL6_JAK_STAT3_SIGNALING genes and continuous AUCell scores in DN cells, with P values adjusted using the false-discovery-rate method. Functional enrichment was performed using the intersection of significantly upregulated genes in AUCell-high cells and detected Hallmark genes. KEGG and Gene Ontology analyses were conducted using clusterProfiler (22), with Benjamini–Hochberg-adjusted P values below 0.05 considered significant (23).

### 2.7 External validation and machine learning-based biomarker screening

An independent bulk gene-expression dataset, GSE30122, comprising 19 DN and 50 control samples, was used for bulk-transcriptomic validation and machine learning-based biomarker screening. Thirty-eight candidate genes derived from the preceding single-cell analyses were evaluated using LASSO, random forest, and Boruta. LASSO logistic regression used a binomial model with alpha = 1, and lambda.min was selected by 10-fold cross-validation (23). Random forest analysis used 2,000 trees and the default classification mtry of six, with the top 15 genes ranked by MeanDecreaseGini retained. Boruta was performed with maxRuns = 500, retaining variables classified as Confirmed. Consensus candidates were defined as genes selected by all three methods. ROC curves were generated using the complete GSE30122 cohort, and AUC values with DeLong 95% confidence intervals were calculated using the pROC package (22–25).

### 2.8 Cell–cell communication analysis

CellChat was used to infer ligand–receptor communication patterns among cells from the DN group (18). Within the DN macrophage population, cells with detectable raw CD36 counts (>0; n = 184) were classified as CD36-detected macrophages, whereas cells with raw CD36 counts equal to zero (n = 294) were classified as CD36-undetected macrophages. The remaining DN cells retained their annotated cell-type identities. The analysis was restricted to the Secreted Signaling category of the human CellChat ligand–receptor database, and inferred interactions involving groups with fewer than 10 cells were excluded (18).

### 2.9 Network-based in silico functional perturbation analysis

Network-based in silico functional perturbation analyses were performed in DN macrophages to evaluate the potential regulatory relevance of CD36. The input feature space comprised detected genes from the HALLMARK_IL6_JAK_STAT3_SIGNALING, HALLMARK_INFLAMMATORY_RESPONSE, and HALLMARK_TNFA_SIGNALING_VIA_NFKB gene sets, together with CD36. CD36 virtual knockout was performed using scTenifoldKnk (26). As a complementary sensitivity analysis, CD36-centered regulatory-edge weights were computationally amplified twofold in the scTenifoldNet-derived macrophage regulatory network (27). Multiple testing was controlled using false-discovery-rate adjustment.

### 2.10 Experimental validation in the DN mouse model

All animal experiments were conducted in accordance with institutional ethics guidelines. Male C57BL/6 mice were randomly assigned to the Control and DN groups, with five animals in each group. Outcome assessment and quantitative analysis were performed in a blinded manner.

DN model mice received a high-fat, high-glucose diet for four weeks, followed by a 12-hour fast and a single intraperitoneal injection of streptozotocin (STZ, 100 mg/kg body weight in 0.1 mol/L citrate buffer, pH 4.5). Control mice were fed a standard chow diet and received an equivalent volume of 0.1 mol/L citrate buffer (pH 4.5) by intraperitoneal injection at the corresponding time point. Two weeks after STZ injection, random blood glucose was measured from tail-vein blood on three consecutive occasions. Mice with random blood glucose ≥16.7 mmol/L in all three measurements were considered successfully diabetic. Qualitative urinary protein was assessed using urine protein test strips. Urinary protein results below 30 mg/L were classified as negative, whereas results at or above 30 mg/L were classified as positive. At the experimental endpoint, mice were anesthetized with isoflurane using 4% for induction and 2% for maintenance until loss of the pedal withdrawal reflex. While under deep anesthesia, mice were euthanized by cervical dislocation. Death was confirmed by cessation of respiration and absence of heartbeat before renal cortical tissue was harvested for histological and molecular analyses.

### 2.11 Histological and immunostaining analyses

Renal sections were stained with hematoxylin–eosin, periodic acid–Schiff, and Masson trichrome. Tubular injury was quantified using the Paller scoring system. Two microscopic fields were evaluated for each mouse, and the mean score across the two fields was used as the animal-level value. For immunohistochemical analysis, renal sections were incubated overnight at 4 °C with an anti-CD36 primary antibody obtained from Wanlei Bio (Cat. No. WL02390; 1:250), followed by a biotinylated secondary antibody and streptavidin–HRP/DAB visualization. The proportion of CD36-positive cells was quantified in three randomly selected microscopic fields per section using ImageJ software (version 1.54t).

For double immunofluorescence staining, renal sections were incubated with an anti-CD36 primary antibody obtained from Huilan Bio (clone H2005, Cat. No. HL20124; 1:100) and an anti-F4/80 primary antibody obtained from Huilan Bio (clone 17-45, Cat. No. ABB00010; 1:200), followed by appropriate fluorescent secondary antibodies. Nuclei were counterstained with DAPI. Three representative non-overlapping microscopic fields were evaluated for each animal under consistent imaging conditions. CD36^+^/F4/80^+^ double-positive cells were counted by an investigator blinded to the experimental grouping, and the mean count across the three fields was used as the animal-level value.

### 2.12 Quantitative real-time PCR

Total RNA was extracted from snap-frozen renal cortical tissue and reverse-transcribed into cDNA (1 μg input RNA). Quantitative real-time PCR was performed in triplicate using SYBR Green master mix. β-actin served as the endogenous reference, and relative expression was calculated by the 2−ΔΔCT method. Primer sequences are provided in **Supplementary Table 2**.

### 2.13 Statistical analysis

Statistical analyses of experimental data were performed using GraphPad Prism 8.0; bioinformatic analyses were conducted in R. Continuous variables are reported as mean ± SD unless otherwise specified. Each animal was treated as an independent biological replicate, and differences between the Control and DN groups were analyzed using a two-sided unpaired Student’s t-test. Between-group comparisons employed the two-tailed Student’s t-test or Mann–Whitney U test as appropriate. Multiple-group comparisons used one-way ANOVA with Tukey’s *post hoc* test. A two-sided P-value < 0.05 was considered statistically significant. AUCell scores were compared between Control and DN cells using two-sided Wilcoxon rank-sum tests, both overall and within individual cell types. No multiple-testing adjustment was applied to the single overall comparison, and P values for cell-type-specific comparisons were reported without adjustment.

## 3 Results

### 3.1 Single-cell landscape of DN reveals heterogeneous epithelial and immune populations

**Figure 1:**
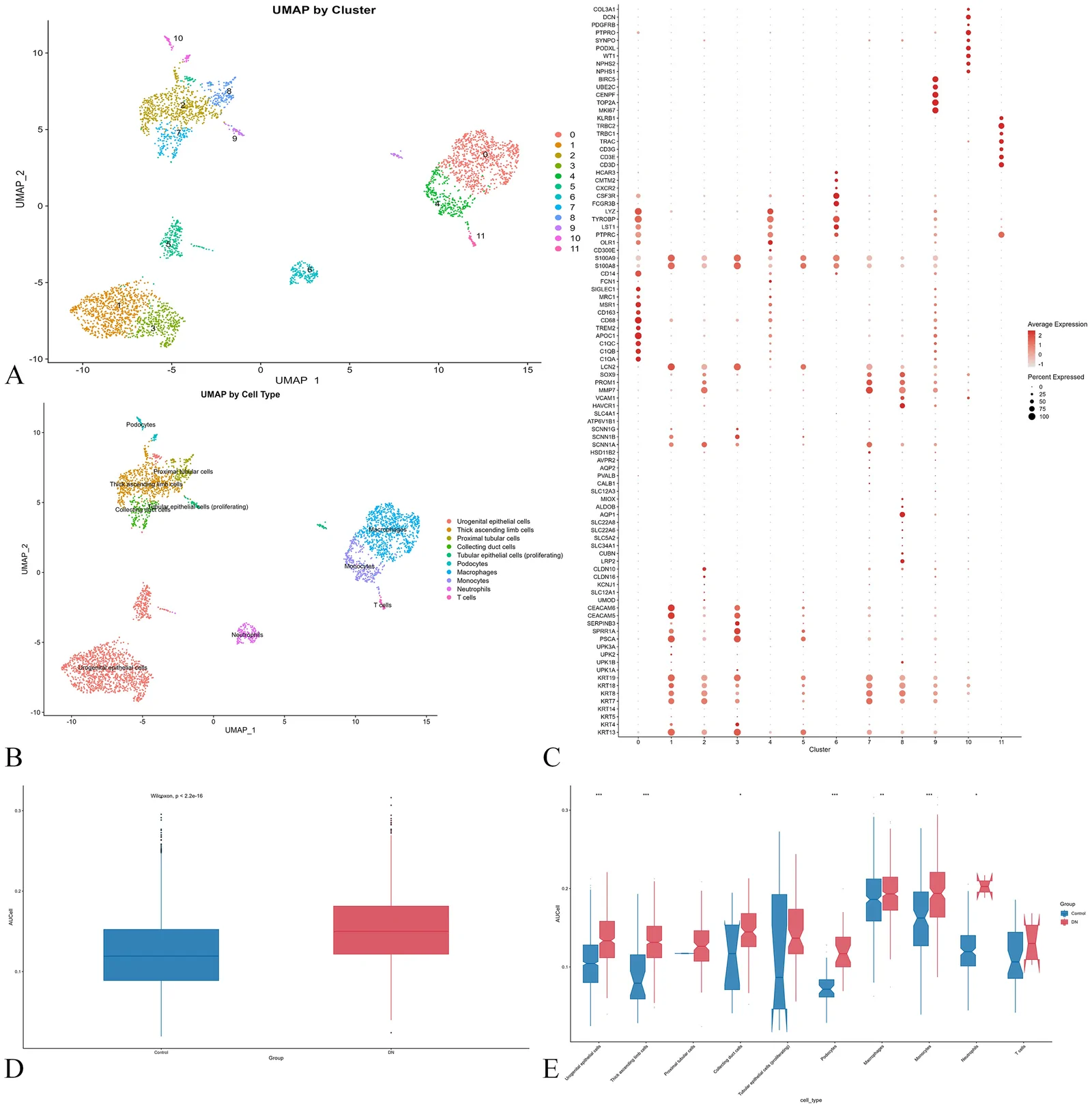
Single-cell landscape, cell-type annotation, and IL6-JAK-STAT3 pathway activity in DN. **(A)** UMAP plot colored by unsupervised cluster identity (0–11). **(B)** UMAP plot annotated by manually assigned cell type. **(C)** Dot plot of canonical marker gene expression across clusters. **(D)** Box plot comparing aggregate AUCell scores between control and DN groups. **(E)** Cell-type-stratified AUCell scores showing between-group differences for each population. two-sided Wilcoxon rank-sum tests; cell-type-specific P values were reported without multiple-testing adjustment. *P < 0.05, **P < 0.01, ***P < 0.001, and ****P < 0.0001.

After quality control, the integrated dataset comprised 3,911 cells from 10 library-level inputs, including 1,797 Control cells from two pooled control libraries and 2,114 DN cells from eight individual DN samples. Detailed library-level cell numbers and cell-type composition by disease group and DN stage are provided in **Supplementary Tables 3**, **4**. Integrated analysis of urine scRNA-seq data from GSE157640 (10 healthy controls) and GSE266146 (8 patients with DN: 4 early-stage [eGFR ≥30 ml/min/1.73 m²] and 4 late-stage [eGFR <30 ml/min/1.73 m²]), followed by Harmony-based batch correction, identified 12 distinct transcriptional clusters (**Figure 1A**). Manual annotation resolved nine major cell populations: B cells, collecting duct cells, macrophages, podocytes, proximal tubular cells, reactive tubular epithelial cells, tubular epithelial cells, proliferating tubular epithelial cells, and urothelial cells (**Figures 1B, C**). Reactive tubular epithelial cells constituted a prominent population in DN compared with controls, suggesting enhanced epithelial stress and injury responses in the disease state. The identification of macrophages as a discrete cluster in urine-derived single-cell data supports the concept that urine provides a non-invasive cellular window into inflammatory activity within the renal parenchyma.

### 3.2 IL6-JAK-STAT3 activity is markedly elevated in DN and enriched in macrophages

AUCell scoring based on the HALLMARK_IL6_JAK_STAT3_SIGNALING gene set revealed a significant global elevation in pathway activity in DN relative to controls (Wilcoxon P < 2.2 × 10^-^¹^6^; **Figure 1D**). Cell-type-stratified analysis demonstrated that macrophages exhibited the highest AUCell scores in the DN condition, with statistically significant differences relative to controls across virtually all annotated cell types (**Figure 1E**). These findings are consistent with the known propensity of macrophages to both produce and respond to IL-6 in inflammatory environments, and identify macrophage-intrinsic JAK-STAT3 activation as a dominant inflammatory program in DN.

### 3.3 Differential expression and enrichment analyses delineate a macrophage-centric inflammatory transcriptome

**Figure 2:**
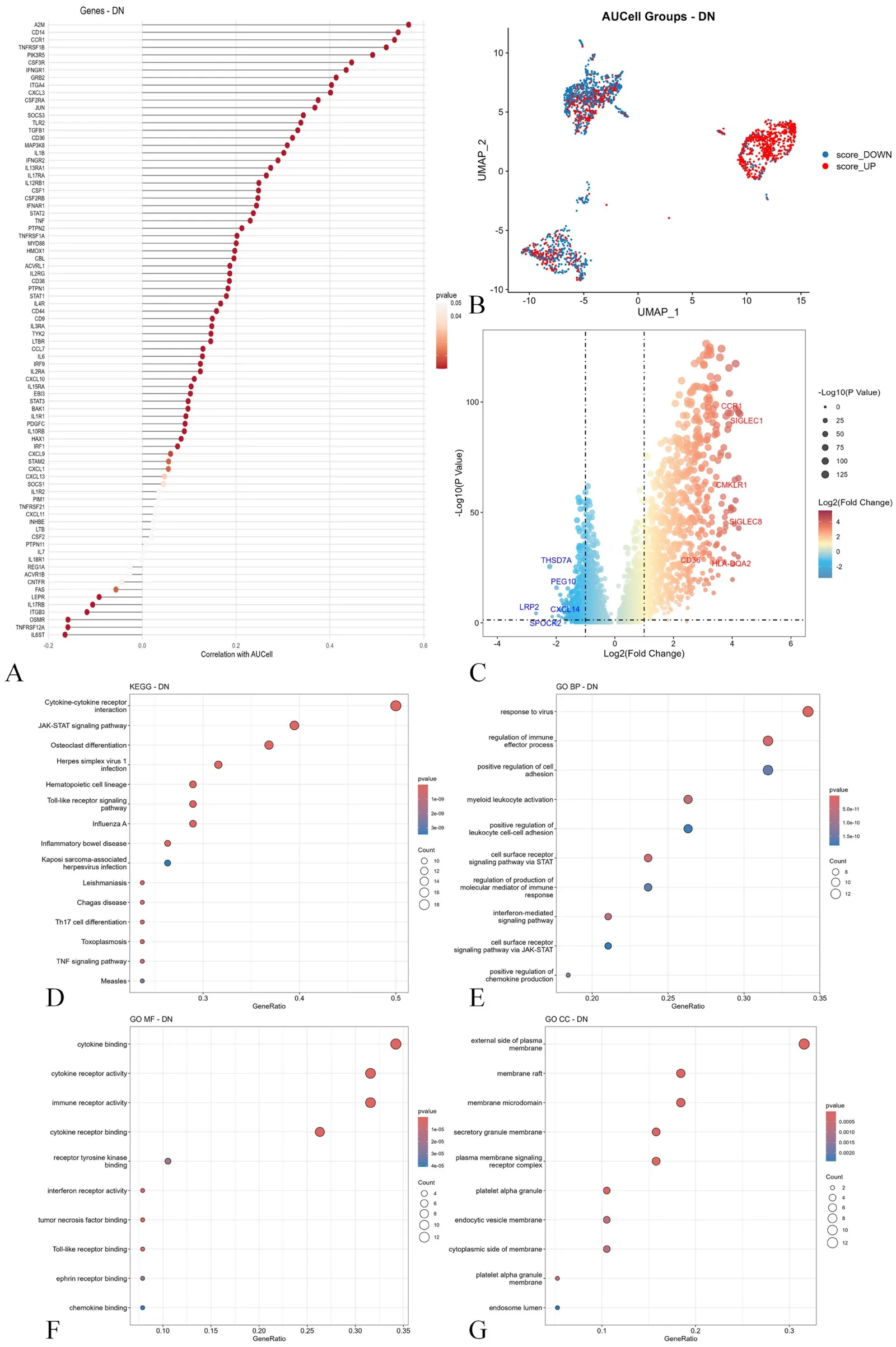
Differential expression and functional enrichment analyses. **(A)** Correlations between gene expression and continuous AUCell scores in DN cells; P values were adjusted using the false-discovery-rate method. **(B)** UMAP distribution of AUCell-high and AUCell-low DN cells defined using the median DN AUCell score. **(C)** Volcano plot of cell-level differentially expressed genes between AUCell-high and AUCell-low DN cells; significance was defined as average log2 fold change >0.25 and Bonferroni-adjusted P<0.05. **(D)** KEGG pathway enrichment analysis. **(E)** Gene Ontology biological process enrichment analysis. **(F)** Gene Ontology molecular function enrichment analysis. **(G)** Gene Ontology cellular component enrichment analysis. Enrichment P values in **(D–G)** were adjusted using the Benjamini–Hochberg method. *P < 0.05, **P < 0.01, ***P < 0.001, and ****P < 0.0001.

Differential expression analysis between AUCell-high and AUCell-low cell populations identified a substantial set of upregulated genes in pathway-active cells, prominently including interferon-stimulated genes, cytokine signaling components, and immune receptor-associated transcripts (**Figures 2A–C**). KEGG enrichment analysis highlighted JAK-STAT signaling, cytokine–cytokine receptor interaction, and hematopoietic cell lineage pathways (**Figure 2D**). GO biological process enrichment revealed significant enrichment of terms related to leukocyte migration, cellular response to interferon, and lymphocyte activation (**Figure 2E**). GO molecular function analysis identified cytokine receptor activity, chemokine binding, and TNF binding as enriched terms (**Figure 2F**), while GO cellular component analysis highlighted plasma membrane-associated compartments (**Figure 2G**)—consistent with the surface receptor-mediated nature of the identified programs.

### 3.4 Multi-algorithm machine learning identifies CD36 as a consensus candidate biomarker

**Figure 3:**
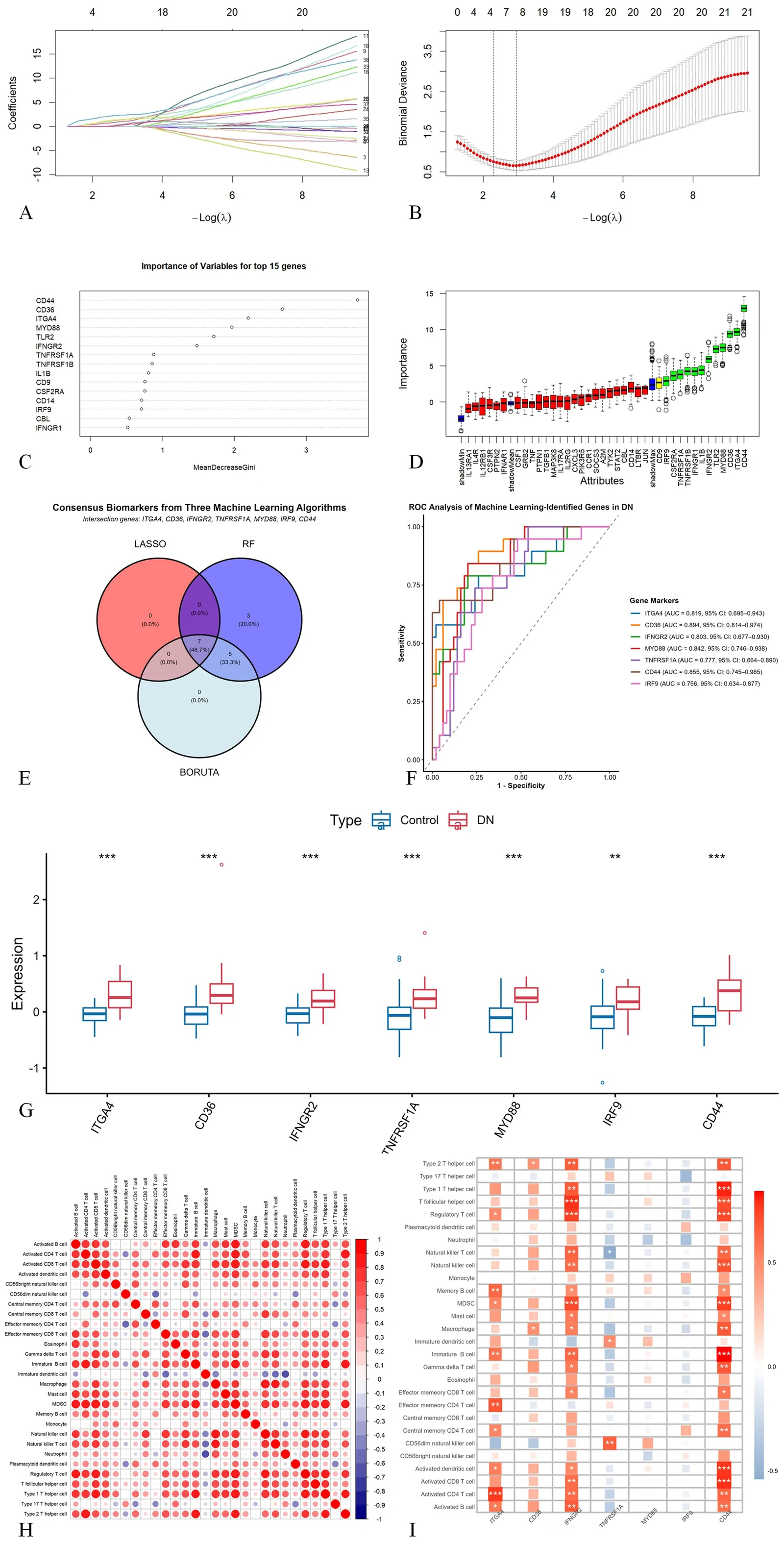
Multi-method feature selection and within-dataset assessment of candidate genes. **(A)** LASSO coefficient profiles of the candidate genes. **(B)** Ten-fold cross-validation for selection of the optimal LASSO penalty parameter. **(C)** The 15 highest-ranked genes identified by random forest according to MeanDecreaseGini. **(D)** Boruta feature-selection results showing the relative importance of candidate variables. **(E)** Intersection of genes retained by LASSO, random forest, and Boruta. Seven consensus genes were identified: ITGA4, CD36, IFNGR2, MYD88, TNFRSF1A, CD44, and IRF9. **(F)** ROC curves of the seven consensus genes in GSE30122. AUC values and DeLong 95% confidence intervals are shown; these results represent within-dataset discrimination. **(G)** Expression distributions of the seven consensus genes between Control and DN samples. **(H)** Correlation matrix of inferred immune-cell populations. Circle size and color indicate the magnitude and direction of the correlation, respectively. **(I)** Associations between the seven consensus genes and inferred immune-cell populations. Color indicates the direction and magnitude of the association, and asterisks denote statistical significance. *P < 0.05, **P < 0.01, ***P < 0.001, and ****P < 0.0001.

LASSO coefficient trajectories and 10-fold cross-validation identified the retained features at the optimal penalty parameter (**Figures 3A, B**). Random forest ranked CD36 among the 15 most important genes according to MeanDecreaseGini (**Figure 3C**), while Boruta classified CD36 as a confirmed relevant feature (**Figure 3D**). The intersection of LASSO, random forest, and Boruta yielded seven consensus genes: ITGA4, CD36, IFNGR2, MYD88, TNFRSF1A, CD44, and IRF9 (**Figure 3E**). In the within-dataset ROC assessment, CD36 achieved an AUC of 0.894 (95% CI, 0.814–0.974; **Figure 3F**), and its expression was significantly higher in DN than in controls (**Figure 3G**). Correlation analysis showed coordinated variation among the inferred immune-cell populations (**Figure 3H**), and several consensus genes, including CD36, were associated with specific immune-cell subsets (**Figure 3I**).

CD36 was prioritized for downstream analysis based on its consistent selection by all three machine-learning methods, favorable discriminatory performance, macrophage-enriched single-cell expression, and biological relevance. In the additional training–validation sensitivity analysis, CD36 remained selected and achieved AUCs of 0.897 in the training set and 0.889 in the held-out internal validation set (**Supplementary Figure 3**).

### 3.5 CD36 is predominantly expressed in macrophages and associated with inferred intercellular communication

**Figure 4:**
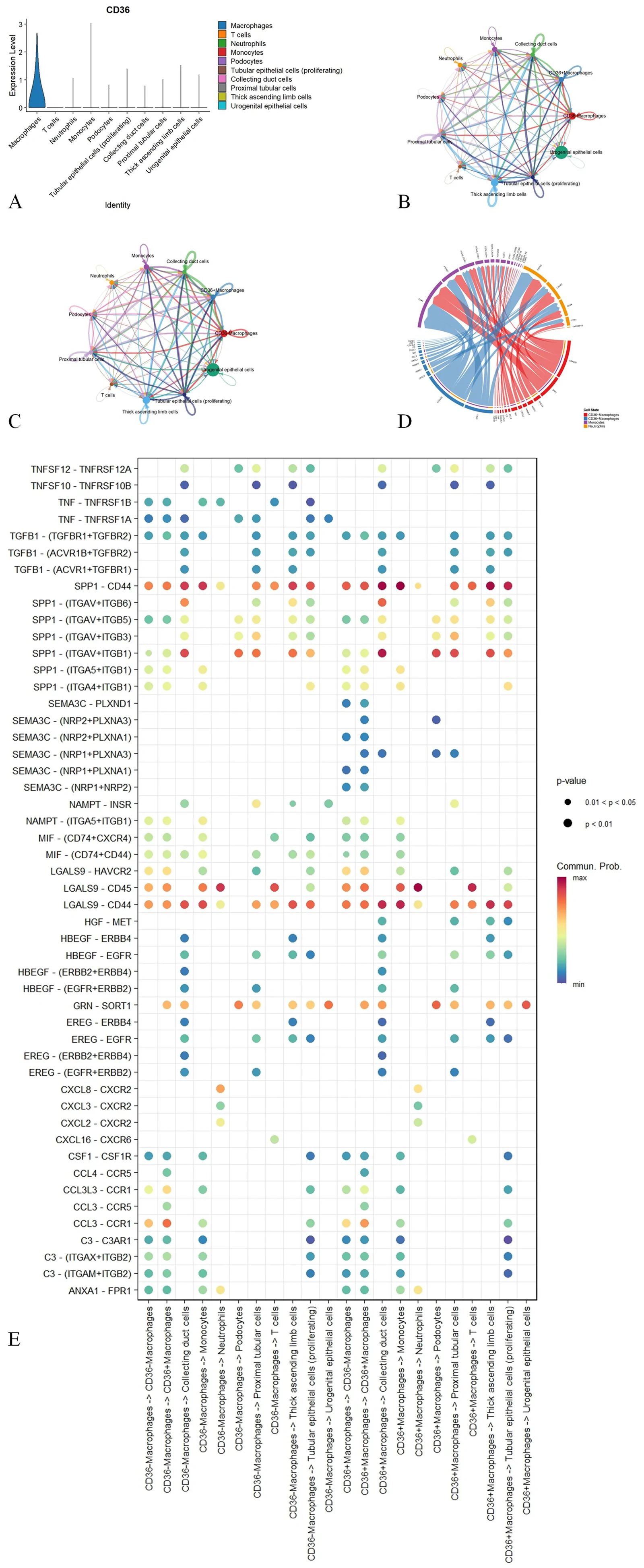
CD36 expression and inferred cell–cell communication in the integrated Control–DN single-cell dataset. **(A)** Violin plot showing CD36 expression across annotated cell types. **(B)** Circle plot showing the number of inferred interactions among all cell groups in the integrated CellChat network. **(C)** Circle plot showing the strength of inferred interactions among all cell groups in the integrated CellChat network. **(D)** Chord diagram showing selected predicted communication pathways among the indicated cell groups, including CD36-detected and CD36-undetected macrophages. **(E)** Bubble plot showing selected predicted ligand–receptor pairs across the indicated sender–receiver cell-group pairs. Dot color represents the inferred communication probability, and dot size indicates statistical significance. Macrophages with raw CD36 counts greater than zero were classified as CD36-detected macrophages (n = 257), whereas macrophages with raw CD36 counts equal to zero were classified as CD36-undetected macrophages (n = 578). All displayed interactions represent computational predictions based on ligand–receptor co-expression.

CD36 expression was enriched in macrophages among the annotated cell populations (**Figure 4A**). CellChat analysis summarized the overall number and strength of inferred interactions across all cell groups in the integrated Control–DN single-cell network (**Figures 4B, C**) (18). Focused analysis of selected communication patterns suggested that CD36-detected and CD36-undetected macrophages may participate as senders and receivers in predicted ligand–receptor networks involving TGF-β/TGFBR, SPP1/CD44, LGALS9/CD44, and CCL/CCR axes (**Figures 4D, E**). An additional analysis restricted to DN cells characterized the corresponding inferred communication patterns within the disease group (**Supplementary Figure 3**). CD36 virtual knockout identified nine genes with significant predicted network perturbations after false-discovery-rate correction, whereas CD36-centered regulatory-edge amplification identified 13 significantly perturbed genes. These findings provided in silico functional support for the potential regulatory relevance of CD36 in DN macrophages (**Supplementary Figure 4**).These findings represent computational predictions based on ligand–receptor co-expression rather than direct functional validation.

### 3.6 Experimental validation confirms increased CD36 expression and macrophage co-localization in DN tissues

**Figure 5:**
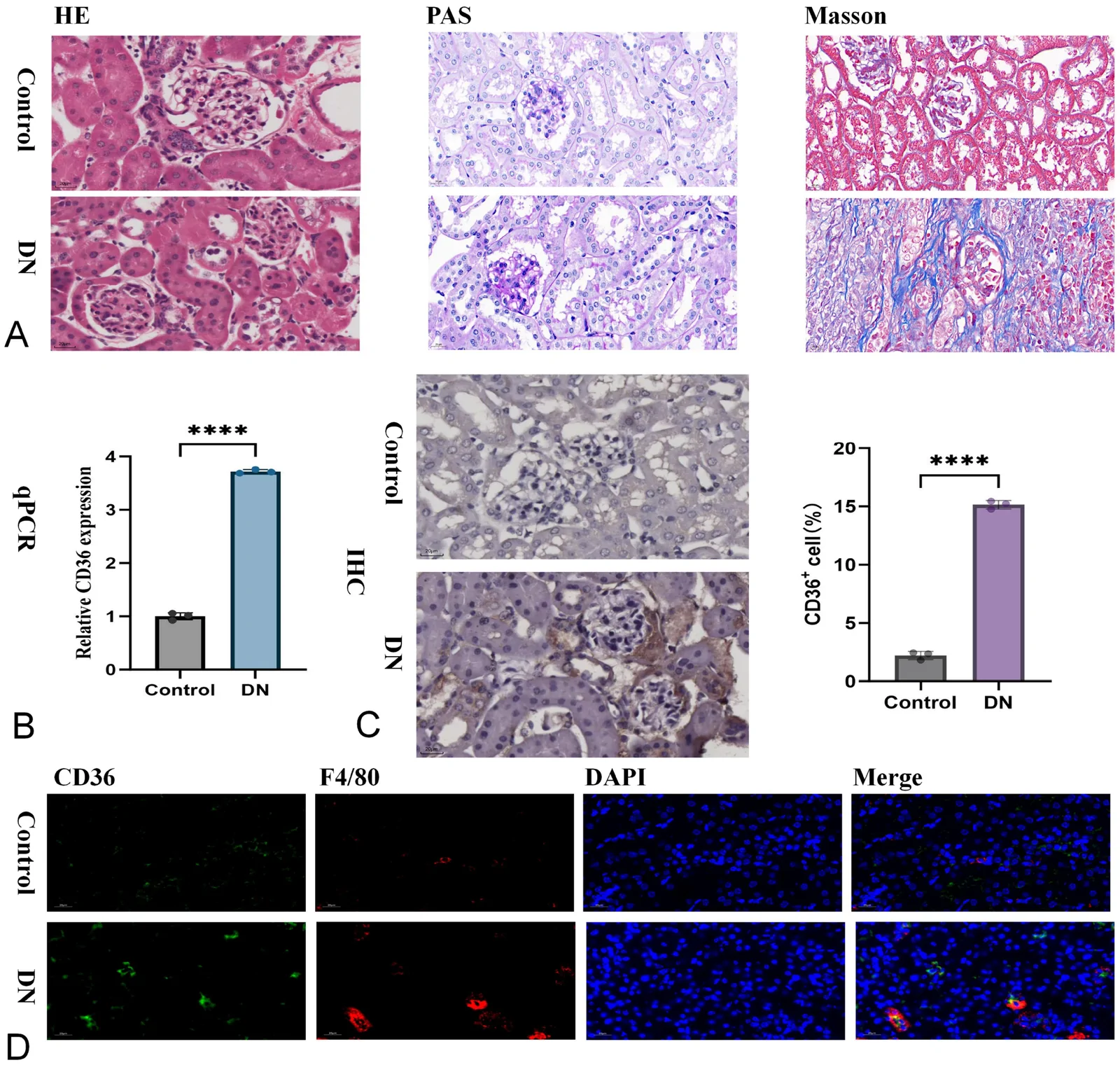
Experimental validation of renal histopathology, CD36 expression, and macrophage co-localization in DN. **(A)** Representative hematoxylin–eosin, periodic acid–Schiff, and Masson trichrome staining of renal tissues from Control and DN mice. **(B)** Quantitative real-time PCR analysis of CD36 mRNA expression in renal tissues. **(C)** Representative immunohistochemical staining of CD36 and quantification of the proportion of CD36-positive cells in renal tissues. **(D)** Representative double-immunofluorescence staining of CD36 (green) and F4/80 (red), with DAPI nuclear counterstaining (blue), showing spatial overlap between CD36 and F4/80 signals. Blinded quantification of CD36^+^/F4/80^+^ double-positive cells is presented in **Supplementary Figure 5**. For quantitative comparisons in **(B)** and **(C)**, each point represents one independent animal (n = 5 mice per group). Data are presented as mean ± SD and were analyzed using two-sided unpaired Student’s t-tests. *P < 0.05, **P < 0.01, ***P < 0.001, and ****P < 0.0001.

The DN group showed significantly higher fasting blood glucose than the Control group (21.22 ± 0.98 versus 4.60 ± 0.37 mmol/L, P < 0.05). Qualitative urinary protein screening was positive in all five DN mice and negative in all five Control mice (P < 0.05). Histological staining of renal tissues from STZ-induced DN mouse demonstrated evident renal injury compared with controls. HE, PAS, and Masson staining showed glomerular and tubular injury, mesangial matrix expansion, and increased extracellular matrix deposition in DN kidneys (**Figure 5A**). qRT-PCR showed significant upregulation of CD36 mRNA in DN renal tissues (**Figure 5B**). Immunohistochemistry further confirmed increased CD36 protein expression in DN kidneys, together with a significantly higher proportion of CD36-positive cells (**Figure 5C**). Double immunofluorescence showed spatial overlap between CD36 and F4/80 signals in renal tissue (**Figure 5D**). Blinded quantitative analysis showed that the mean number of CD36^+^/F4/80^+^ double-positive cells per field was significantly higher in the DN group than in the Control group (9.73 ± 1.77 versus 3.27 ± 1.72; P = 0.0004; **Supplementary Figure 5**), supporting increased spatial association between CD36 expression and F4/80-positive macrophages in DN.

## 4 Discussion

The present study integrates urine-derived single-cell transcriptomics, multi-algorithm machine learning, and CellChat-based intercellular communication analysis to characterize the macrophage-associated inflammatory landscape of DN and identify CD36 as a macrophage-enriched candidate biomarker. Importantly, this study is distinguished from prior single-modality transcriptomic analyses by three converging features: first, the use of urine-derived scRNA-seq as a non-invasive approach that enables clinical scalability without the ethical and procedural constraints of kidney biopsy; second, the consensus biomarker selection strategy requiring concordance across three independent machine learning algorithms (15, 28), which substantially reduces dataset-specific false discovery; and third, the integration of CellChat-based communication modeling to move from biomarker identification toward a mechanistic understanding of communication patterns associated with CD36-detected macrophages within the renal microenvironment. Together, these elements provide a more robust and clinically relevant framework for identifying macrophage-associated biomarkers in DN compared to conventional single-modality approaches (15, 18–20, 28).

Our finding that macrophages in the urine single-cell transcriptome of DN patients exhibit the highest IL6-JAK-STAT3 AUCell activity scores is consistent with, and extends, a body of literature implicating macrophage IL-6 signaling in progressive renal injury. IL-6 trans-signaling—mediated by soluble IL-6 receptor shedding and downstream JAK1/TYK2-STAT3 activation—promotes macrophage survival, enhances monocyte recruitment, and induces adhesion molecule expression on tubular cells, creating a self-reinforcing inflammatory circuit. The observation that pathway activity is elevated not only in macrophages but also, to a lesser degree, in tubular epithelial cells and podocytes supports a model of paracrine amplification in which macrophage-derived IL-6 reprograms resident renal cells. Future spatial transcriptomic analyses in kidney biopsy tissue would be valuable to determine whether the macrophage–tubular proximity observed histologically correlates with the communication interactions predicted by our CellChat models (3, 4, 6, 8, 29, 30).

Although several consensus genes also showed favorable diagnostic signals, CD36 was prioritized because it combined stable machine-learning support, favorable diagnostic performance, macrophage-enriched single-cell expression, and biological relevance to lipid-associated inflammation. The identification of CD36 as a consensus machine learning biomarker—retained by LASSO, random forest, and Boruta across independent datasets—directly addresses a persistent challenge in transcriptomic biomarker research: the tendency for individually identified features to be dataset-specific and non-reproducible. By requiring concordance across three algorithms with fundamentally different selection strategies (penalized regression, ensemble tree-based classification, and permutation-based relevance testing), our approach substantially reduces spurious associations. CD36’s AUC of 0.894 in the external validation cohort represents strong individual-gene diagnostic performance, and its macrophage-enriched expression pattern provides a biologically coherent mechanistic rationale: elevated CD36 reflects the expansion and transcriptional activation of a macrophage population that is characteristic of the DN inflammatory state (23, 25).

CD36 was prioritized on the basis of convergent evidence rather than a single selection criterion. It was retained by all three feature-selection methods, showed the highest apparent within-dataset AUC among the seven consensus candidates, and exhibited macrophage-enriched expression. Its continued selection and favorable performance in the additional training–validation sensitivity analysis further supported its prioritization. Together with the biological involvement of CD36 in lipid uptake, macrophage activation, and inflammatory injury, these findings supported its selection for downstream characterization in DN.

CellChat predicted TGF-β/TGFBR, SPP1/CD44, LGALS9/CD44, and CCL/CCR ligand–receptor co-expression patterns involving CD36-detected macrophages. The integrated analysis characterized the overall communication network across Control and DN cells, whereas the DN-restricted analysis examined the corresponding predicted communication patterns within the disease group. These findings suggest that CD36-detected macrophages may participate in intercellular communication involving these signaling axes in DN. Complementary in silico perturbation analyses indicated that the inferred macrophage regulatory network was sensitive to computational alterations of CD36-centered relationships, while tissue-level experiments supported increased renal CD36 expression and its spatial association with F4/80-positive macrophages. However, CD36 was used only to define the macrophage subgroups in the CellChat analysis and was not modeled as the direct ligand or receptor of these signaling axes. Moreover, the in silico perturbation and tissue-level analyses provide separate supportive evidence but do not directly validate the CellChat-predicted ligand–receptor interactions. Therefore, these findings support an association between CD36 and macrophage-associated alterations in DN but do not establish direct mechanistic causality.

The use of urine-derived single-cell transcriptomics warrants specific comment. Conventional kidney biopsy-based profiling is limited by procedural risk, sampling variability, and ethical constraints that restrict large-cohort application. Our study demonstrates that urine-derived scRNA-seq can resolve macrophage transcriptional states with sufficient granularity to enable AUCell pathway scoring and cell-communication inference—capabilities previously demonstrated primarily in biopsy-derived datasets. Previous urine cell studies in lupus nephritis, IgA nephropathy, and acute kidney injury have established the biological relevance of this approach; the present work extends it to DN and provides proof-of-concept for non-invasive immune cell profiling in diabetic renal disease (15, 19, 20, 28, 31, 32).

Despite these limitations, the present findings have clear translational implications. CD36 may warrant further therapeutic investigation, and experimental antagonism of CD36 has been shown to attenuate kidney inflammation and fibrosis and to slow chronic kidney disease progression in preclinical models (13, 33), supporting further investigation of CD36-targeted interventions in DN. Given that urine-derived cells can be obtained entirely non-invasively, CD36 expression in urinary macrophages may represent a candidate biomarker requiring prospective validation for monitoring inflammatory activity in DN in clinical settings, which warrants prospective validation in larger cohorts with standardized urine cell collection protocols (11, 13, 33).

Taken together, these findings support a model in which CD36-positive macrophages represent a functionally distinct inflammatory state that integrates lipid metabolism, cytokine signaling, and intercellular communication in DN—a conceptual framework that may guide both biomarker development and the design of targeted anti-inflammatory interventions in DN (7, 12, 13).

In summary, the present study identified IL6–JAK–STAT3-enriched macrophage states and prioritized CD36 as a macrophage-enriched candidate associated with favorable within-dataset discrimination and predicted intercellular communication patterns in DN. These findings support further investigation of CD36 but do not establish it as an independently validated diagnostic biomarker, therapeutic target, or causal regulator.

### 4.1 Limitation

Several limitations merit acknowledgment. First, the human scRNA-seq analysis was limited by the small number of publicly available urine-derived samples, which may not fully represent kidney tissue, and residual batch effects after dataset integration cannot be excluded. Because the Control and DN data came from separate studies and the Control libraries were pooled, residual dataset-related confounding cannot be excluded; therefore, the findings should be considered exploratory. Dedicated doublet-calling algorithms were also not applied. Second, the AUCell-high versus AUCell-low differential-expression analysis was performed at the cell level without pseudobulk aggregation. Third, CellChat provides inference based on ligand–receptor co-expression rather than direct functional validation. No experimental CD36 knockdown or pharmacological inhibition was performed; therefore, its biological and therapeutic effects require further experimental validation. Finally, the STZ mouse model and the limited *in vivo* cohort size (n = 5 per group) may limit generalizability, warranting validation in larger independent cohorts and complementary disease models.

## Data Availability

Publicly available datasets were analyzed in this study. This data can be found here: https://www-ncbi-nlm-nih-gov.brum.beds.ac.uk/geo/.
