# Supplementary material for: Integrated single-cell transcriptomic and machine-learning analyses identify a CD36-associated macrophage state in diabetic nephropathy
Source: Front Immunol. 2026 Aug 20;17:1868468. doi: 10.3389/fimmu.2026.1868468 (PMC13538110; doi:10.3389/fimmu.2026.1868468)
Supplement: Supplementary file 1 [file Table1.docx]

**Supplementary Table S1. Final cluster assignments and canonical markers**

| **Cluster** | **Final cell type** | **Canonical markers used for annotation** |
| --- | --- | --- |
| 0 | Macrophages | C1QA, C1QB, C1QC, APOC1, TREM2, CD68, CD163, MSR1, MRC1, SIGLEC1, LST1, TYROBP, LYZ |
| 1 | Urogenital epithelial cells | KRT4, KRT7, KRT8, KRT13, KRT17, KRT18, KRT19, UPK1A, UPK1B, UPK2, UPK3A |
| 2 | Thick ascending limb cells | UMOD, SLC12A1, KCNJ1, CLDN16, CLDN10 |
| 3 | Urogenital epithelial cells | KRT4, KRT7, KRT8, KRT13, KRT17, KRT18, KRT19, UPK1A, UPK1B, UPK2, UPK3A |
| 4 | Monocytes | FCN1, CD14, S100A8, S100A9, CD300E, OLR1, LST1, TYROBP, LYZ |
| 5 | Urogenital epithelial cells | EPCAM, KRT4, KRT7, KRT8, KRT13, KRT17, KRT18, KRT19, UPK1A, UPK1B, UPK2, UPK3A |
| 6 | Neutrophils | FCGR3B, CSF3R, CXCR2, CMTM2, HCAR3, S100A8, S100A9, TYROBP, LYZ |
| 7 | Collecting duct cells | AQP2, AVPR2, HSD11B2, SCNN1A, SCNN1B, SCNN1G, ATP6V1B1, SLC4A1, EPCAM |
| 8 | Proximal tubular cells | LRP2, CUBN, SLC34A1, SLC5A2, SLC22A6, SLC22A8, AQP1, ALDOB, MIOX, DPP4 |
| 9 | Proliferating tubular epithelial cells | EPCAM, KRT8, KRT18, MKI67, TOP2A, CENPF, UBE2C, BIRC5, AURKB, CCNA2 |
| 10 | Podocytes | NPHS1, NPHS2, WT1, PODXL, SYNPO, PTPRO, MAFB, TCF21 |
| 11 | T cells | CD3D, CD3E, CD3G, TRAC, TRBC1, TRBC2, CD2, KLRB1 |
