# Supplementary material for: Integrated single-cell transcriptomic and machine-learning analyses identify a CD36-associated macrophage state in diabetic nephropathy
Source: Front Immunol. 2026 Aug 20;17:1868468. doi: 10.3389/fimmu.2026.1868468 (PMC13538110; doi:10.3389/fimmu.2026.1868468)
Supplement: Supplementary file 2 [file Table2.docx]

**Supplementary Table S2. Primer Sequences Used for qRT-PCR**

| Gene | Forward Primer (5′→3′) | Reverse Primer (5′→3′) |
| --- | --- | --- |
| CD36 | GAAGCCAATCTTACGGTCCTG | CACATTTCAGAAGGCGGCAA |
| β-actin | GTCCACCCGCGAGTACAAC | GGATGCCTCTCTTGCTCTGG |
