# Supplementary material for: Integrated single-cell transcriptomic and machine-learning analyses identify a CD36-associated macrophage state in diabetic nephropathy
Source: Front Immunol. 2026 Aug 20;17:1868468. doi: 10.3389/fimmu.2026.1868468 (PMC13538110; doi:10.3389/fimmu.2026.1868468)
Supplement: Supplementary file 3 [file Table3.docx]

**Table S3. Library-level retained cell numbers and cell-type composition of the integrated single-cell dataset**

| **Public accession** | **Library/sample ID** | **Group/stage** | **Sample structure** | **Retained cells** |
| --- | --- | --- | --- | --- |
| GSE157640 | GSE157640_control_pool | Control | Pooled from 5 healthy donors | 918 |
| GSE165396 | GSE165396_control_pool | Control | Pooled from 12 healthy donors | 879 |
| GSE266146 | early1 | Early DN | Individual sample | 28 |
| GSE266146 | early2 | Early DN | Individual sample | 56 |
| GSE266146 | early3 | Early DN | Individual sample | 131 |
| GSE266146 | early4 | Early DN | Individual sample | 211 |
| GSE266146 | adv1 | Advanced DN | Individual sample | 157 |
| GSE266146 | adv2 | Advanced DN | Individual sample | 667 |
| GSE266146 | adv3 | Advanced DN | Individual sample | 260 |
| GSE266146 | adv4 | Advanced DN | Individual sample | 604 |
| **Control total** | **2 pooled libraries** | **Control** | **17 healthy donors** | **1,797** |
| **DN total** | **8 individual samples** | **DN** | **4 early and 4 advanced samples** | **2,114** |
| **Overall** | **10 library-level inputs** | **-** | **-** | **3,911** |
