# Supplementary material for: Integrated single-cell transcriptomic and machine-learning analyses identify a CD36-associated macrophage state in diabetic nephropathy
Source: Front Immunol. 2026 Aug 20;17:1868468. doi: 10.3389/fimmu.2026.1868468 (PMC13538110; doi:10.3389/fimmu.2026.1868468)
Supplement: Supplementary file 4 [file Table4.docx]

**Table S4. Retained cells by final annotated cell type**

| **Final annotated cell type** | **Control** | **Early DN** | **Advanced DN** | **DN total** | **Overall** |
| --- | --- | --- | --- | --- | --- |
| Urogenital epithelial cells | 998 | 152 | 308 | 460 | 1,458 |
| Thick ascending limb cells | 33 | 72 | 506 | 578 | 611 |
| Proximal tubular cells | 1 | 13 | 143 | 156 | 157 |
| Collecting duct cells | 11 | 21 | 128 | 149 | 160 |
| Tubular epithelial cells (proliferating) | 12 | 8 | 59 | 67 | 79 |
| Podocytes | 45 | 14 | 16 | 30 | 75 |
| Macrophages | 357 | 92 | 386 | 478 | 835 |
| Monocytes | 114 | 52 | 138 | 190 | 304 |
| Neutrophils | 185 | 2 | 0 | 2 | 187 |
| T cells | 41 | 0 | 4 | 4 | 45 |
| **Total** | **1,797** | **426** | **1,688** | **2,114** | **3,911** |
