# Supplementary material for: Integrated single-cell transcriptomic and machine-learning analyses identify a CD36-associated macrophage state in diabetic nephropathy
Source: Front Immunol. 2026 Aug 20;17:1868468. doi: 10.3389/fimmu.2026.1868468 (PMC13538110; doi:10.3389/fimmu.2026.1868468)
Supplement: Supplementary file 5 [file Table5.docx]

Table S5. Phenotypic characterization of the STZ-induced diabetic mouse model

| Variable | Control (n = 5) | DN (n = 5) | P value |
| --- | --- | --- | --- |
| Fasting blood glucose, mmol/L | 4.60 ± 0.37 | 21.22 ± 0.98 | <0.05 |
| Positive qualitative urinary protein, n/N | 0/5 | 5/5 | <0.05 |
| Paller tubular injury score | 0.00 ± 0.00 | 5.40 ± 0.19 | <0.05 |
