# Supplementary material for: Integrated single-cell transcriptomic and machine-learning analyses identify a CD36-associated macrophage state in diabetic nephropathy
Source: Front Immunol. 2026 Aug 20;17:1868468. doi: 10.3389/fimmu.2026.1868468 (PMC13538110; doi:10.3389/fimmu.2026.1868468)
Supplement: Supplementary file 6 [file Table6.docx]

To examine the integrated representation, Harmony-corrected UMAP plots were visualized by sample identity, dataset of origin, and disease group (Supplementary Figure S1). Samples from the three source datasets contributed to the integrated transcriptional representation, while the control and DN groups showed both overlapping and partially distinct distributions. These plots were used as a qualitative assessment of sample contributions, dataset-associated structure, and disease-group distribution after Harmony integration.

Supplementary Figure S1. Distribution of sample sources and disease groups in the Harmony-integrated single-cell representation.

(A) Harmony-corrected UMAP colored by DN sample identity and control-dataset source. The eight DN samples from GSE266146 are displayed individually, including four early-DN samples and four advanced-DN samples, whereas control cells are grouped according to their source datasets, GSE157640 and GSE165396. (B) Harmony-corrected UMAP colored by disease group, including Control and DN. Each point represents one cell. These plots were used to qualitatively assess the contributions and distributions of the available sample or dataset categories and disease groups after Harmony integration.

Supplementary Figure S2. Canonical marker expression across annotated single-cell clusters.

Dot size represents the percentage of cells expressing each gene, and color intensity indicates the scaled average expression level. Marker-expression patterns support the final annotation of clusters 0–11.

**Supplementary Figure S3. Internal training–validation sensitivity analysis of machine-learning-based candidate selection in GSE30122.**

(A) LASSO coefficient profiles generated using the training set. (B) Ten-fold cross-validation curve for selection of the LASSO penalty parameter in the training set. (C) The 15 highest-ranked genes identified by random forest according to MeanDecreaseGini. (D) Boruta feature-selection results showing the relative importance of candidate variables. (E) Boruta importance trajectories across 500 classifier runs. (F) Intersection of genes retained by LASSO, random forest, and Boruta in the training set. Six consensus genes were identified: ITGA4, CXCL3, CD36, IFNGR2, MYD88, and CD44. (G) ROC curves for the six consensus genes in the training set.
(H) ROC curves for the same genes in the held-out internal validation set.

**Figure S4. DN-restricted analysis of CD36 expression and inferred cell–cell communication.**
**(A)** Violin plot showing CD36 expression across annotated cell types within the DN group. **(B)** Circle plot showing the number of inferred interactions among all cell groups in the DN-restricted CellChat network. **(C)** Circle plot showing the strength of inferred interactions among all cell groups in the DN-restricted CellChat network. **(D)** Chord diagram showing selected predicted communication pathways among the indicated cell groups, including CD36-detected and CD36-undetected macrophages. **(E)** Bubble plot showing selected predicted ligand–receptor pairs across the indicated sender–receiver cell-group pairs within the DN group. Dot color represents the inferred communication probability, and dot size indicates statistical significance. Macrophages with raw CD36 counts greater than zero were classified as CD36-detected macrophages (n = 184), whereas macrophages with raw CD36 counts equal to zero were classified as CD36-undetected macrophages (n = 294). All displayed interactions represent computational predictions based on ligand–receptor co-expression.

**Figure S5. Network-based in silico functional perturbation analysis of CD36 in DN macrophages.**

(A) Significance plot showing predicted network perturbations following CD36 virtual knockout using scTenifoldKnk. (B) Top 20 genes ranked by predicted perturbation magnitude following CD36 virtual knockout. (C) Significance plot showing predicted network perturbations following twofold amplification of CD36-centered regulatory-edge weights. (D) Top 20 genes ranked by predicted perturbation magnitude following CD36-centered edge amplification. The dashed horizontal line indicates an FDR-adjusted P value of 0.05. Perturbation scores represent predicted changes in the inferred regulatory-network state rather than experimentally measured gene-expression fold changes.

Figure S6. Quantification of CD36⁺/F4/80⁺ double-positive cells in renal tissue.

Three non-overlapping microscopic fields were evaluated per animal, and the mean count was used as the animal-level value. Each point represents one independent animal (n = 5 per group). Data are presented as mean ± SD. Differences were analyzed using a two-sided unpaired Student’s t-test. ***P < 0.001.
